# Supplementary material for: Women’s representation as authors of retracted papers in the biomedical sciences
Source: PLoS One. 2023 May 3;18(5):e0284403. doi: 10.1371/journal.pone.0284403 (PMC10155963; doi:10.1371/journal.pone.0284403)
Supplement: S1 Table — (DOCX) [file pone.0284403.s001.docx]

**Table S1: Women’s representation among authors of retracted papers**

| First author (80% accuracy) | Number | Percentage [95% CI] |
| --- | --- | --- |
| Women | 5613 | 27.4 [26.8 to 28.0] |
| Men | 14876 |  |
| First author (60% accuracy) |  |  |
| Women | 7650 | 26.9 [26.3 to 27.4] |
| Men | 20831 |  |
| Last author (80% accuracy) |  |  |
| Women | 4802 | 23.5 [22.9 to 24.1] |
| Men | 15611 |  |
| Last author (60% accuracy) |  |  |
| Women | 6702 | 23.6 [23.2 to 24.1] |
| Men | 21634 |  |
