# Supplementary material for: Women’s representation as authors of retracted papers in the biomedical sciences
Source: PLoS One. 2023 May 3;18(5):e0284403. doi: 10.1371/journal.pone.0284403 (PMC10155963; doi:10.1371/journal.pone.0284403)
Supplement: S2 Table — (DOCX) [file pone.0284403.s002.docx]

**Table S2: Women’s representation among authors of retracted papers over time (from 1970)**

| Time | First author | N retractions | Last author | N retractions |
| --- | --- | --- | --- | --- |
| 1971-80 | 5.1 | 39 | 17.5 | 40 |
| 1981-90 | 12.0 | 166 | 11.0 | 163 |
| 1991-00 | 13.3 | 670 | 9.5 | 666 |
| 2001-10 | 23.9 | 5,737 | 18.3 | 5,630 |
| 2011-22 | 29.8 | 13,873 | 26.5 | 13,910 |
