# Supplementary material for: Women’s representation as authors of retracted papers in the biomedical sciences
Source: PLoS One. 2023 May 3;18(5):e0284403. doi: 10.1371/journal.pone.0284403 (PMC10155963; doi:10.1371/journal.pone.0284403)
Supplement: Attachment — Submitted filename: rebuttal-letter-v3.docx [file pone.0284403.s003.docx]

**PONE-D-22-33690:** **Women’s representation as authors of retracted papers in the biomedical sciences**

**Answers to reviewers’ comments**

Reviewer #1: Overall, this is an interesting project, that possess relevant questions, but a larger literature review is needed. The general over or under representation of women in retracted papers can only be accounted if compared with the gender composition among all authors. The differences between the different retraction types are interesting and do not suffer from the problem mentioned above. Nevertheless, much more work on the different rejection types, their motivations and main actors, together with the authorship distribution of tasks and potential responsibilities is needed to make this a valuable contribution. Below some more detailed comments.

We thank Reviewer #1 very much for their comments.

Abstract:

The abstract needs some re-writing. An introductory paragraph that contextualizes the research would be helpful. Results should only be a summarized sentence of the abstract, explaining the general conclusions.

We added a couple of sentences as background. We did not change the results because the journal allows an abstract of up to 300 words and we considered that it would be useful for readers to have a more detailed description of the results than a single sentence.

Page 2, Abstract

Women are under-represented among authors of scientific papers. Although the number of retractions has been rising over the past few decades, gender differences among authors of retracted papers remain poorly understood. Therefore, this studied investigated gender differences in authorship of retracted papers in biomedical sciences available on RetractionWatch.

Introduction:

The introduction is rather short, and the framing of this project in the previous literature on gender inequalities in science and retracted articles is missing.

We added two paragraphs to the introduction:

Page 3, line 41

Peer reviewed papers are retracted when they are considered an invalid source of scientific knowledge and hence should be completely removed from the scientific record. According to the Committee on Publication Ethics (COPE)(1), papers should be retracted in the following circumstances: (i) clear evidence for misconduct or honest error; (ii) duplicate publication without proper reference; (iii) plagiarism, and (iv) unethical research. Despite the proposal of the COPE, there is no consensual definition of retraction and the terms retraction, correction, withdrawal, removal, expression of concern, erratum, and corrigendum are often used interchangeably. Although the reasons for retraction are not always clear, they are often provided by retraction notices. According to a previous study, the most common reason for retraction is research misconduct (fabrication, falsification, or plagiarism), which accounts for about 60% of retractions.(2) Most retractions (about 60%) are initiated by author(s), followed by editors (about 20%), publishers (about 15%), journals (about 5%), and institutions (less than 1%).(3)

Although retractions are rare, their absolute number and relative proportion of published papers, have been increasing over the past two decades.(4, 5) In the biomedical literature, the number and proportion of retractions by year increased from 1980 to 2014 (particularly after 2004) and declined after 2015.(4) Current estimations for the proportion of retractions are about 4 in 10,000. However, the proportion of retractions varies by publisher, with high impact journals having the highest retraction rates.(3) The cause for this recent rise in retractions is not clear but it seems to be the effect of growing scientific integrity, rather than growing scientific misconduct.(6) Evidence suggests that researchers and journal editors have become more aware of and more proactive about scientific misconduct, without a true increase in cases of fraud.

Methods

- The data limitations and potential biases should be considered.

We expanded the section on the limitations in the discussion as we did not consider it would be appropriate to discuss this in the methods:

Page 7, line 186

Our study is the first to have investigated retractions in relation to gender using a large sample of biomedical papers published over four decades. However, the large number of papers meant that we had to base gender determination by a software tool based on authors’ first names. Although this software has been found to be the most reliable at estimating gender, it is not completely accurate, especially for names using non-Latin alphabets. In addition, this approach fails to account for non-binary gender identification as it classifies authors as women or men.(9) In addition, although RetractionWatch is the largest database of retracted papers, it does not capture all retractions as not all publishers clearly label or publicise papers they have retracted or disclose the underlying reasons.

- What does each rejection reason imply? Which are the potential motivations between the different rejection types, and how this is appears on the literature? Are different rejection types potentially more related with an authorship position? For example, is there a relation between data manipulation (and rejections related with concerns about data) and first authors? And between issues related with misconduct in authorship or issues with editors and last authors? What does the literature say about this?

Although we understand the reviewer’s questions, there is no published literature addressing those questions. We believe the sections added to the introduction (see answer above) summarise the current knowledge about retractions.

- L72 “and test whether these percentages were significantly different from 50%”. This hypothesis is highly problematic. It is well known in the literature that women are underrepresented in science. If the authors want to test gender differences on retraction, they should control by the composition of authors in biomedical sciences between 1971 and 2022 (i.e. the composition at the population level). This might be the most important problem of the article and needs to be address somehow (a limitation statement would not fix this problem). Further controls could be made, for example accounting for the gender composition on the exact set of journals considered, or subfields. If the authors do not have access to a bibliometric database that allows them to compute the gender composition at the authors or paper level, then maybe the hypothesis of underrepresentation of women in the retracted papers should be removed from the analysis. As the literature shows (and the authors noted in the discussion), the proportion of women authors is normally around 30%, which is the same order of magnitude the authors found on retractions. The differences between 27% and 30% can only be significant on comparable datasets.

We added a sensitivity analysis using women’s representation as first and last author of biomedical papers reported in recent literature. We tested whether their representation as authors of retractions was significantly different from their representation as authors of papers overall using a threshold of 40% for first author and 30% for last author. We explained this in the methods and reported the findings in the results:

Page 4, line 109

As a sensitivity analysis, we tested whether women’s representation as authors of retractions was comparable to their representation as authors of papers overall. Based on previous literature showing the underrepresentation of women in authorship of biomedical papers, we also tested whether those percentages were significantly different from 40% for first authors and 30% for last authors.(11, 12)

Page 5, line 127

Sensitivity analyses comparing women’s representation as authors of retractions with their representation as authors of papers overall were comparable to the main analyses. The percentage of women as authors of retractions was significantly lower than their representation as authors of biomedical papers for both first and last author (p<0.0001).

Results

- The analysis of results needs more work. This is an interesting dataset, but much more work should be done with it. What does the evolution over time say? How does it compare with the gender composition of the field? Which is the relation between first/last author and the different rejection types?

We explored our findings in greater detail in the discussion as follows:

Page 5, line 145

Women’s underrepresentation among first and last authors of retracted papers is slightly lower than women’s representation among first and last authors of biomedical papers in general. Although there is substantial variation between biomedical fields, women have consistently represented about 30-40% of first authors and 25-30% of last authors.(7, 11, 12) These trends over time are also broadly comparable to general trends in authorship in biomedical sciences.(13) For instance, in medical journals, there was a 4.2% increase in the proportion of women authors between 2008 and 2018, with a larger increase for women as last authors than as first authors (7.8% versus 3.6%, respectively).(11) We found a greater increase in women as first than last authors, with the percentage of women as first authors remaining larger than last authors over time. This is in keeping with the greater representation of women as first than last authors of biomedical papers overall.(11) The increase in women’s representation as authors of retractions is also in line with the rise in the number and proportion of retractions, which seems to be related to greater awareness and commitment to research integrity than an actual increase in fraudulent research.(6)

- Also, if the authors have the information, an analysis by disciplines would be interesting.

We do not have this information.

Reviewer #2: From a bibliometric perspective the paper can be improved by:

- Information on the distribution of the gender of authors in biomedical scientific papers

We expanded this paragraph in the discussion as follows:

Page 5, line 146

Although there is substantial variation between biomedical fields, women have consistently represented about 30-40% of first authors and 25-30% of last authors.(7, 11, 12) These trends over time are also broadly comparable to general trends in authorship in biomedical sciences.(13) For instance, in medical journals, there was a 4.2% increase in the proportion of women authors between 2008 and 2018, with a larger increase for women as last authors than as first authors (7.8% versus 3.6%, respectively).(11)

- Information on the distribution of papers by the number of its authors in biomedical scientific papers

Please see answer to the comment above.

- Discussion on the complexities of author position in a scientific papers and the role of cultural and geographical factors in assigning authors order. For example, Suzetta Burrows & Mary Moore (2011), Journal of Electronic Resources in Medical Libraries, 8: 2,155-168) states that "there are still no universal policies to guide author order in biomedical research publication bylines. Misunderstandings about the placement of a particular author in a sequence of co-authors are common. This, together with changes in indexing policies"

This is a valid point, but it is beyond the scope of this paper, which does not aim to explore authorship position but representation of women among authors. We added this to the limitations as follows:

Page 7, line 194

There is still no consensus about the importance of order of authors in biomedical papers and this may have influenced our findings of women’s representation for different authorship positions.(21)

From a sociological perspective the paper can be improved by revising the title as it suggests the study approaches the representation authors of retracted papers have on women: in social sciences and humanities, studies on gender representations follow in-depth and qualitative methodologies. The study follows the participation of women as authors (first and last) of retracted papers in biomedical sciences.

We amended the title as follows:

Women’s representation as authors of retracted papers in the biomedical sciences

The inclusion of a more descriptive analysis of the gender distribution in the 35.635 papers considered, on the distribution of gender in authorship collaborations in the sample (see for example papers on gender homophile in scientific collaboration).

We expanded this section in the discussion as follows:

Page 5, line 145

Women’s underrepresentation among first and last authors of retracted papers is slightly lower than women’s representation among first and last authors of biomedical papers in general. Although there is substantial variation between biomedical fields, women have consistently represented about 30-40% of first authors and 25-30% of last authors.(7, 11, 12) The trends over time are also broadly comparable to general trends in authorship.(13) For instance, in medical journals, there was a 4.2% increase in the proportion of women authors between 2008 and 2018, with a larger increase for women as last authors than as first authors (7.8% versus 3.6%, respectively).(11) We found a greater increase in women as first than last authors, with the percentage of women as first authors remaining larger than last authors over time. This is in keeping with the greater representation of women as first than last authors of biomedical papers overall.(11) The increase in women’s representation as authors of retractions is also in line with the rise in the number and proportion of retractions, which seems to be related to greater awareness and commitment to research integrity than an actual increase in fraudulent research.(6)

Authors should address the limitations of the study, a discussion on the generalizability of the empirical results should be included, particularly for strengthening the conclusion that "Gender equality could improve research integrity in biomedical sciences".

We added a sentence to the limitations as follows:

Page 7, line 196

It is uncertain how generalisable our findings are to other fields beyond biomedical sciences, as practices may vary regarding authorship and retraction criteria between scientific fields.
