# Supplementary material for: Women’s representation as authors of retracted papers in the biomedical sciences
Source: PLoS One. 2023 May 3;18(5):e0284403. doi: 10.1371/journal.pone.0284403 (PMC10155963; doi:10.1371/journal.pone.0284403)
Supplement: Attachment — Submitted filename: rebuttal-letter-v6.docx [file pone.0284403.s004.docx]

**PONE-D-22-33690:** **Women’s representation as authors of retracted papers in the biomedical sciences**

**Additional Editor Comments:**
Dear Authors:
This revised version has addressed the majority of reviewers concerns regarding contextualization of current evidence about gender differences in retracted scientific papers in biomedical sciences. You have also provided information necessary for obtaining access to third-party data according to PLOSONE Data Availability Statement.
I am pointing out minor issues (previous comments included) that require your attention before considering your paper ready for publication:

1. Please consider further explanation about the systematic selection and adjudication process of included papers to show reproducibility: As your study deals with a sample of retracted papers, please provide methods used during paper selection. If not providing a selection flowchart, please provide number of papers per reason of exclusion in your main text.

We were provided with a bespoke dataset of papers retracted in biomedical sciences by Retraction Watch. We added a brief explanation about this source and cited it as recommended by the source. We also included an additional explanation in the limitations. We did not exclude any papers from this dataset. Papers were only excluded due to inability to predict authors based on first names of first or last authors. We clarified this in the manuscript:

Page 4, line 83

We used a database of retracted papers curated by RetractionWatch [10]. This is the largest, most comprehensive database of retracted papers across multiple scientific fields. It was launched in 2010 and is continuously updated by pulling retractions from existing databases, such as PubMed, or publishers’ sites. We were provided with a bespoke dataset comprising 35,635 papers in biomedical sciences published between January 1971 and September 2022. No papers were excluded. We extracted data for the first and last author of each paper. We did not include other authors as their contribution and responsibility for the content of papers in biomedical sciences is variable and sometimes minimal. The first author should be the person who contributed most to the work, including writing of the manuscript, whilst the last author is commonly the senior author. We inferred their gender based on their first names using Gender-API software [11]. We accepted gender predictions when the accuracy was estimated to be at least 80% and performed sensitivity analysis for an accuracy of 60% or over. This meant that 14,786 and 15,222 papers were excluded from the main analysis of first and last author, respectively (7,154 and 7,299 papers were excluded from the sensitivity analysis for first and last author).

Page 10, line 249

In addition, although RetractionWatch is the largest database of retracted papers, it does not capture all retractions as not all publishers clearly label or publicise papers they have retracted or disclose the underlying reasons. In addition, the database was only started in 2010.

2. Please address Reviewer #2 concerns about limitations and conclusions based on your findings.

We have responded to Reviewer #2 concerns.

3. Please complete thorough grammar and typos revision on your manuscript.
We have done this.

Reviewers' comments:

Reviewer #1: I consider that the paper was much improved after the review, and it is ready for publication. As a suggestion, the figure 2 could be improved with the new information of the overall proportion of women as first and last authors in the discipline, maybe with two vertical lines, that would help the reader as a benchmark.

We have included those lines as suggested by the reviewer and added an explanation in the figure legend. Please see Figure 2.

Reviewer #2: The comments have been addressed but some concerns about the paper raised by the reviewers’ comments remain solved. Providing context on the overall distribution of gender among authors of the selected sample would benefit the paper and its argument, as well as more detail on why looking only at the gender of first and last authors makes sense.
We clarified in the methods that this study is not based on a “selected sample”. Rather, it is based on the full database of retracted papers in biomedical sciences provided by Retraction Watch. Studies investigating gender inequalities have typically focused on first and last authors because, in biomedical sciences, these are the guarantors of the paper. The first author is usually the lead author who carried out the research and the last author is usually the most senior author who supervised the research. The contribution and responsibility of middle authors is highly variable and the number of authors differs substantially between papers. Therefore, we considered this analysis would not be relevant for this study and focused on first and last authors. We clarified this in the methods:

Page 4, line 87

We extracted data for the first and last author of each paper. We did not include other authors as their contribution and responsibility for the content of papers in biomedical sciences is variable and sometimes minimal. The first author should be the person who contributed most to the work, including writing of the manuscript, whilst the last author is commonly the senior author.

The large database we used only includes retracted papers, whilst, as far as we can find, there is also no reference to compare women’s representation among authors of retracted papers and papers in general. What we can do is to compare it against the first and last authorship by females in medical journals per se. We have added this as supplementary analysis and added the reference lines to Figure 2 to make even more explicit that women’s representation among authors of retracted papers needs to consider women’s overall representation as authors of biomedical papers. Please see Figure 2.

In the Abstract, stating that "this studied investigate" makes no sense. The paper needs a thorough revision of grammar and possible typos.

Thank-you for pointing this out. We have thoroughly newly reviewed the manuscript and corrected all typos.
